# Spatial Distribution and Characteristics of Protein Content and Composition in Japonica Rice Grains: Implications for Sake Quality

**DOI:** 10.1186/s12284-024-00708-w

**Published:** 2024-04-12

**Authors:** Kei Takahashi, Hiromi Kohno, Masaki Okuda

**Affiliations:** National Research Institute of Brewing, 3-7-1 Kagamiyama, Higashi-hiroshima, Hiroshima 739-0046 Japan

**Keywords:** Glutelin, Prolamin, Rice grain, Seed storage protein, Protein body type-II (PB-II), Crude protein, Endosperm, Rice-polishing ratio, *Yamadanishiki*. *Gohyakumangoku*

## Abstract

The quantity and composition of rice proteins play a crucial role in determining taste quality of *sake*, Japanese rice wine. However, the spatial distribution of proteins within rice grains, especially in endosperm tissue, and the differences between rice varieties remain unclear. Here, we analyzed the crude protein contents and composition ratios of table (*Nipponbare* and *Koshihikari*) and genuine sake rice varieties (*Yamadanishiki*, *Gohyakumangoku*, *Dewasansan*, *Dewanosato*, and *Yumenokaori*) to elucidate their spatial distribution within the Japonica rice grain endosperm. Seven sake rice varieties were polished over five harvest years using a brewer’s rice-polishing machine. We obtained fractions at 90–70% (the outermost endosperm fraction), 70–50%, 50–30%, and 30–0% (the central region of the endosperm fraction). *Yamadanishiki* and *Dewanosato* exhibited considerably lower crude protein contents than the other cultivars. After applying SDS-PAGE, the protein composition, comprising glutelin/total protein (G/TP), prolamin/TP (P/TP), and G/P ratios of these fractions was determined. In white rice (at a 90% rice-polishing ratio), the average ratio of the major protein composition was G/TP 41%, P/TP 21%, and G/P ratios of 1.97. *Gohyakumangoku* and *Yamadanishiki* had higher G/TP ratio, while *Dewanosato* had a lower value. Despite having lower crude protein contents, *Yamadanishiki* and *Dewanosato* exhibited significantly varying G/TP ratios. The G/TP ratio markedly varied among rice varieties, particularly in the rice grains’ central region. The 50–30% fraction had the highest P/TP ratio among all tested rice varieties, suggesting spatial differences in P/TP within rice grains. *Koshihikari* had the lowest P/TP ratio. In addition, the 50–30% fraction had the lowest G/P ratio among all tested rice varieties, with *Gohyakumangoku* having the highest G/P ratio. *Dewanosato* had the lowest G/P value, and this value significantly differed from that of *Yamadanishiki* in the 30–0% fraction. We found substantial differences in protein composition within distinct spatial regions of rice grains, and larger differences among rice varieties were observed in the rice grain’s central region.

**Supplementary Information:**

The online version contains supplementary material available at 10.1186/s12284-024-00708-w.

## Background

The protein content in brown or polished white rice is a key indicator of table rice palatability (Kondo and Nozoe 1993; Martin and Fitzgerald 2002; Matsue et al. 1996). For genuine sake rice, a Japonica rice variety specifically bred and used for Japanese sake brewing, the protein content in rice grains is also considered a crucial indicator of rice quality (Iemura et al. 1995, 1999a, b; Takahashi et al. 2021a; Wakai et al. 1997). Typically, the crude protein content in white rice with a 70% rice-polishing ratio (the ratio [w/w] of polished rice to original brown rice) has been the standard measure because rice for sake brewing is highly polished compared to table rice (Kizaki et al. 1991; Takahashi et al. 2021a). In sake brewing, the brewmaster initiates the production of *koji*, a rice mold that grows *Aspergillus oryzae* on and in polished rice grains (Kanauchi 2013). While the fungi generate numerous proteases and peptidases for protein degradation into oligopeptides and amino acids, these enzymes also enzymatically digest rice proteins into nitrogen-containing compounds such as oligopeptides and amino acids within the *koji*. As the sake brewing process proceeds according to the multiple parallel fermentation manner, in which the *koji*-produced enzymes are active in the main mash known as “*moromi*,*”* proteases and peptidases can continue to degrade rice proteins. Notably, the resulting nitrogen-containing compounds are subsequently incorporated into sake yeast and utilized as energy sources and cellular constituents. However, the protease and peptidase would remain active even in the middle and later stages of mash when the stage sake yeast population has reached a saturation point and its growth has ceased. Therefore, a considerable quantity of nitrogen-containing compounds should remain in the final sake product.

Some sake are brewed using highly polished rice because an excessive amount of amino acids in sake can impart an unpleasant, unsophisticated taste, especially for *ginjo* sake (Iemura et al. 1996; Iwano et al. 2005; Takahashi and Kohno 2016). In contrast, the presence of extremely few assimilable nitrogen compounds in the mash due to the usage of “low-glutelin rice” as an ingredient presents challenges in controlling yeast growth effectively, which may result in the production of sake with undesirable flavors (Mizuma and Furukawa 2004; Mizuma et al. 2002).

In rice endosperms, the majority of proteins are accumulated in small particles known as “protein bodies” located between large starch crystals. These rice protein bodies can be divided into two types. Protein body type-I (PB-I), which originates from the rough endoplasmic reticulum (ER), forms a spherical shape with a diameter of 1–2 μm and accumulates various types of prolamin gene products (Mitsukawa et al. 1999; Nagamine et al. 2011; Saito et al. 2012; Tanaka et al. 1980). An *in silico* database search has identified 34 prolamin genes in *O. sativa* (Xu and Messing 2009), with more than 20 estimated to exist in flawless form in rice (Saito et al. 2012). Most of these prolamin genes are subclassified of 13-kDa prolamin, while minor types include 10-kDa and 16-kDa prolamins (Saito et al. 2012). These prolamin proteins form multiple layers within the spherical PB, with each layer comprising the same gene products. For example, the core comprises cysteine-rich 10-kDa prolamin, but the outermost layer comprises cysteine-poor 13-kDa prolamin (Nagamine et al. 2011; Saito et al. 2012). Owing to the physical properties of densely packed PB-I, prolamin exhibits resistance to digestive enzymes such as proteases and peptidases. Protein body type-II (PB-II), which is derived from the vacuole, forms irregularly shaped granules with a diameter of 3–4 μm and accumulates various types of glutelins and 26-kDa alpha-globulin (Kumamaru et al. 2010; Takaiwa et al. 1987; Yamagata et al. 1982). Glutelin is initially synthesized as a precursor approximately 50–55-kDa in polypeptide length (Takahashi et al. 2019; Yamagata et al. 1982). Subsequently, it is cleaved by a vacuolar processing enzyme (Kumamaru et al. 2010; Wang et al. 2009) into a 30–35-kDa acidic subunit and a 20-kDa basic subunit (Takahashi et al. 2019; Yamagata et al. 1982). Glutelin is the product of several glutelin genes (Kusaba et al. 2003; Masumura et al. 1989a; Mitsukawa et al. 1998; Qu et al. 2002; Takaiwa et al. 1987, 1991; Takaiwa and Oono 1991) that are categorized into four glutelin subfamilies (GluA, GluB, GluC, and GluD) based on their amino acid sequence homology (Kawakatsu and Takaiwa 2010; Kawakatsu et al. 2008). The localization and distribution of these glutelin gene products in rice grains can markedly differ based on specific glutelin genes (Takahashi et al. 2019). For instance, GluA exhibits a strong localization in the outer region of the endosperm, with a much weaker presence in the central region, extending its localization to the aleurone layer and even into the embryo, but GluC displays a uniform distribution throughout the endosperm. The localization pattern of glutelin proteins in the endosperm remains largely consistent across seven Japonica rice varieties, including popular sake rice used in practical sake brewing. A rice variety specific distribution feature is also indicated. Proteases and peptidases readily degrade glutelin, unlike prolamin, leading to the release of amino acids and oligopeptides during sake brewing. Thus the quantity and/or proportion of glutelin and prolamin relative to total protein (TP) can be crucial factors in rice processing, particularly in sake brewing.

Studies on the PB-II/PB-I ratio in white rice grains have been reported previously. The glutelin (acidic and basic subunits)/prolamin (10-, 13-, and 16-kDa prolamin) ratio in white rice, including table and sake rice, at a 70% rice-polishing ratio was 2.42 (Kizaki et al. 1991) or 2.67 (Kizaki et al. 1993) using a designated baseline on the chromatogram chart. This ratio was also reported to be 2.2 using the same extraction and analytical methods as Kizaki et al., except for the use of rice variety and another densitometer (Furukawa et al. 2000). Ashida et al. reported that the PB-II (acidic and basic subunits of glutelin, and alpha-globulin)/PB-I ratio in *Yamadanishiki*, the most popular sake rice variety, at a 90% rice-polishing ratio was 2.08, and the glutelin (acidic and basic subunits)/prolamin (13-, and 16-kDa prolamin) ratio was 1.77 (Ashida et al. 2013). As these reports have shown, the glutelin/prolamin ratio considerably varies in 1.77–2.67 depending on factors such as the methods used, including protein extraction and denaturing, the rice-polishing ratio, and rice variety. Despite the importance of the quantity and/or proportion of glutelin and prolamin to TP for sake brewing, the spatial distribution of nitrogen compounds in rice grains, especially in endosperm tissue, and the differences between rice varieties remain unclarified. In this study, we analyzed the crude protein content and composition ratio of sake rice in selected varieties to elucidate the spatial distribution within the rice grain endosperm.

## Materials and Methods

### Plant Materials

Seven cultivars of brown rice were obtained from the Society for the Studies of Brewer’s Rice and harvested in 2009, 2010, 2011, 2013 and 2014. These included *Koshihikari* (Chiba, Chiba), *Nipponbare* (NRIB, Higashi-Hiroshima, Hiroshima), *Yamadanishiki* (NRIB, Higashi-Hiroshima, Hiroshima), *Gohyakumangoku* (Kitakata, Fukushima), *Dewasansan* (Sakata, Yamagata), *Dewanosato* (Higashi-Okitama, Yamagata), and *Yumenokaori* (Kitakata, Fukushima). All rice plants were grown in a paddy field at each location according to the growth conditions required for each rice variety.

### Preparation of Fractionated Rice Powder from Endosperms

Fractionated rice powder was obtained as described previously (Takahashi et al. 2019). A home rice polisher was used to polish brown rice to 90% (w/w) of the original weight. The resulting polished rice to 90% rice-polishing ratio (the ratio [w/w] of polished rice to original brown rice) was sequentially polished to 70, 50, and 30% by a grain test mill HS-4 using whetstone roll#60 and a roll mesh (Chiyoda Engineering, Inc., Hiroshima, Japan). Fractionated rice powder from four different spatial rice grain*—*90–70%, 70–50%, 50–30%, and 30–0% of rice-polishing ratio*—*was obtained as described previously (Takahashi et al. 2019).

### Crude Protein Contents

Fractionated rice powder and brown rice powder from different crop years (*n* = 5) were analyzed using an elemental analyzer (Vario EL III, Elementar, Langenselbold, Germany). Nitrogen content was quantified, and the coefficient for rice protein, 5.95, was multiplied to calculate the crude protein content. The crude protein content of each rice variety was indicated as a dry matter. Briefly, rice moisture was determined as follows: Rice flour (1.5 g) was sampled into an aluminum dish with a cover. The samples were completely dried by heating in a drying container at 135ºC for 2 h. After 10-min cooling in a desiccator, immediately their weight was determined. The difference in rice powder weight measured before and after drying was taken as the moisture value.

### SDS-PAGE and Protein Composition Analysis

A previous report outlined the process of protein extraction from rice powder and the method of moderate denaturation to achieve higher resolution SDS-PAGE results (Takahashi et al. 2019). SDS-PAGE was performed using 15% polyacrylamide gels (ATTO, Tokyo, Japan) with protein-size markers (Bio-Rad, Hercules, MA, USA). After separation, the proteins were stained with 0.1% Coomassie Brilliant Blue (CBB) R-250 (Nacalai tesque, Kyoto, Japan). The gel was destained for at least 3 h before being scanned directly. Quantity One software ver. 4.6.9. (Bio-Rad) was used to determine the intensity of TP intensity in a lane, glutelin acidic subunit, glutelin basic subunit, and prolamin. The ratios of glutelin (G) / TP, prolamin (P) / TP, and other proteins / TP were calculated as follows:

$$ \text{G}/\text{T}\text{P} = (\text{g}\text{l}\text{u}\text{t}\text{e}\text{l}\text{i}\text{n} \,\text{a}\text{c}\text{i}\text{d}\text{i}\text{c} \,\text{s}\text{u}\text{b}\text{u}\text{n}\text{i}\text{t}\hspace{0.17em}+\hspace{0.17em}\text{g}\text{l}\text{u}\text{t}\text{e}\text{l}\text{i}\text{n}\, \text{b}\text{a}\text{s}\text{i}\text{c} \,\text{s}\text{u}\text{b}\text{u}\text{n}\text{i}\text{t}) / \text{T}\text{P}$$

$$ \text{P}/\text{T}\text{P}\hspace{0.17em}=\hspace{0.17em}\text{p}\text{r}\text{o}\text{l}\text{a}\text{m}\text{i}\text{n} / \text{T}\text{P}$$

$$ (\text{T}\text{P}-\text{G}-\text{P})/\text{T}\text{P} = \frac{\left(\begin{aligned}&\text{T}\text{P}-\text{g}\text{l}\text{u}\text{t}\text{e}\text{l}\text{i}\text{n}\,\text{a}\text{c}\text{i}\text{d}\text{i}\text{c}\,\text{s}\text{u}\text{b}\text{u}\text{n}\text{i}\text{t}\\&-\text{g}\text{l}\text{u}\text{t}\text{e}\text{l}\text{i}\text{n}\,\text{b}\text{a}\text{s}\text{i}\text{c}\,\text{s}\text{u}\text{b}\text{u}\text{n}\text{i}\text{t}-\text{p}\text{r}\text{o}\text{l}\text{a}\text{m}\text{i}\text{n}\end{aligned}\right)}{\text{T}\text{P}}$$

### Statistical Analysis

Statistical differences in crude protein and protein composition in rice-polishing ratio and rice variety were analyzed with analysis of variance (ANOVA) and Tukey–Kramer HSD test using JMP software ver.10.0.2 (SAS Institute, Cary, NC, USA).

## Results

### Crude Protein Contents

**Fig. 1:**
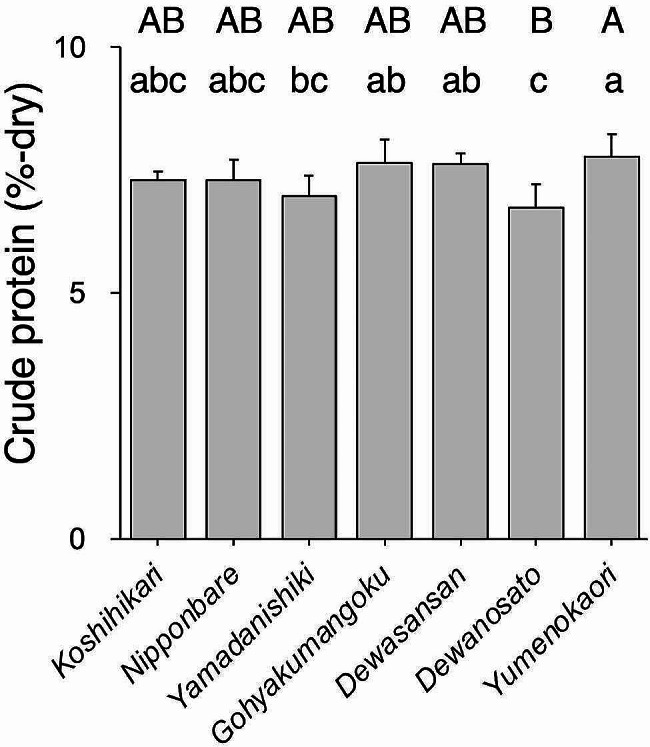
Crude protein content of brown rice The average crude protein contents in brown rice of different rice varieties (*n* = 5, as crop year) are shown. The crude protein content of each rice variety is indicated as dry matter. Data containing different capital and small letters indicate significance by Tukey–Kramer HSD test at *p* < 0.01 and *p* < 0.05, respectively

**Fig. 2:**
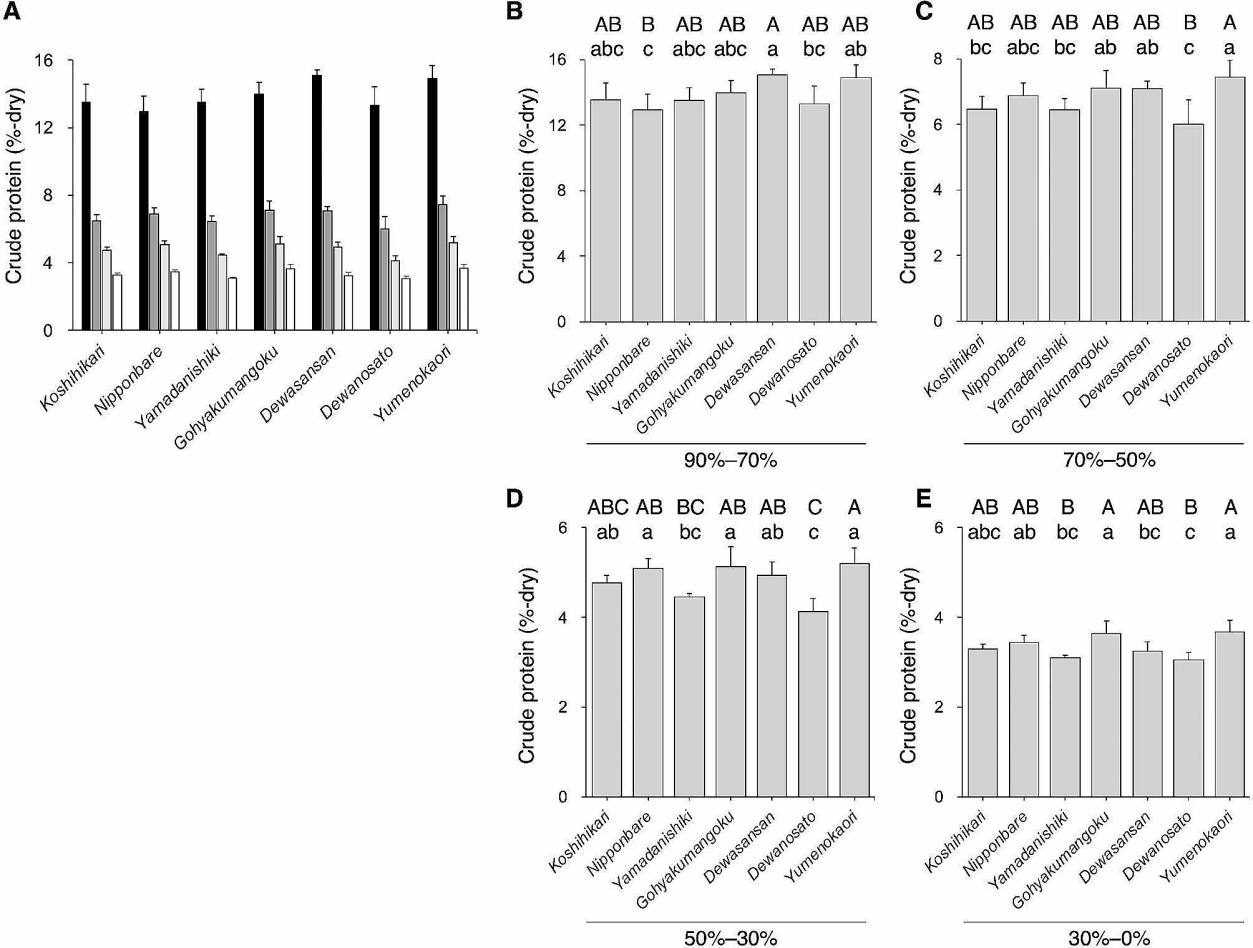
Crude protein content of polished rice. The average crude protein contents in fractionated rice powders of different rice varieties (*n* = 5, as crop year) are shown. The crude protein content of each rice variety is indicated as dry matter. (A) Crude protein contents of polished rice with rice-polishing ratio of 90–70% (closed bars), 70–50% (dark gray bars), 50–30% (light gray bars), and 30–0% (opened bars). Crude protein contents of the ratios of 90–70% (B), 70–50% (C), 50–30% (D), and 30–0% (E). Data containing different capital and small letters indicate significance by Tukey–Kramer HSD test at *p* < 0.01 and *p* < 0.05, respectively

To determine the crude protein content of whole brown rice used for practical sake production, nitrogen levels were quantified through elemental analysis. *Yumenokaori* had higher crude protein content than *Yamadanishiki* and *Dewanosato* (Fig. 1). To gain insight into the spatial distribution of protein in the rice grains, the crude protein content of sequentially polished rice flours was analyzed. The rice grains’ outer region with a rice-polishing ratio of 90–70%, contained over 13% of the crude protein content in all tested rice varieties, whereas it decreased to half in the rice-polishing ratio of 70–50% (Fig. 2A). In the rice grains’ central region, with a rice-polishing ratio of 30–0%, the crude protein content was below 4%. The crude protein content was the lowest in *Nipponbare* among the tested rice varieties at a rice-polishing ratio of 90–70%, and *Dewanosato* contained the lowest content in the other powder fractions (Fig. 2B–E). In the fractions of 70–50% through 30–0%, the rice variety with the lowest crude protein content was *Dewanosato*, followed by *Yamadanishiki* (Fig. 2C–E). This finding corroborated a previous report in which white rice with a 70% rice-polishing ratio of *Dewanosato* and *Yamadanishiki* exhibited markedly lower crude protein content than other sake rice harvested in 2002–2019 (Takahashi et al. 2021a).

### Protein Composition

**Fig. 3:**
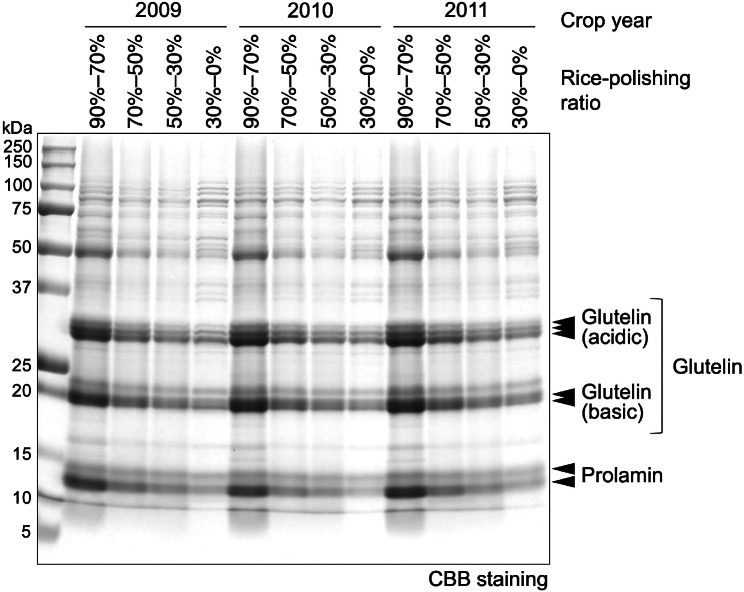
SDS-PAGE and protein composition analysis of fractionated rice powders. A representative SDS-PAGE result from fractionated rice powders with rice-polishing ratios of 90–70%, 70–50%, 50–30%, and 30–0% from three different crop years (2009, 2010, 2011). Each lane contains the same weight of rice powder

To determine the proportion of major protein components in rice grains, TP, glutelin acidic subunit, glutelin acidic subunit, and prolamin were quantified after SDS-PAGE. A typical SDS-PAGE result is shown in Fig. 3, as reported previously (Takahashi et al. 2019). The intensity of the stained protein in each fraction was almost the same for TP by densitometry after SDS-PAGE and for crude protein content by elemental analysis (data not shown). Given the inherent difficulty in achieving a perfect comparison of quantified textual data of stained proteins for more than two independently stained gels even when using molecular markers as external standards, we calculated the molecular ratio of proteins in each lane to evade this problem: (1) G/TP, (2) prolamin to total protein (P/TP), (3) glutelin/prolamin (G/P), and (4) proteins other than glutelin and prolamin (TP–G–P). The average value of the protein ratio with seven rice varieties in white rice with a 90% rice-polishing ratio was as follows: G/TP ratio, 41%; P/TP ratio, 21%; TP–G–P, 38%; G/P, 1.97. Although the TP–G–P value contained 26-kDa globulin and minor seed storage proteins such as proglutelin bands presenting in 50–55-kDa, 16-kDa prolamin, and 10-kDa prolamin, 38% of the TP–G–P protein quantity markedly exceeded previously reported values.

### The Ratio of Glutelin/Total Protein

**Fig. 4:**
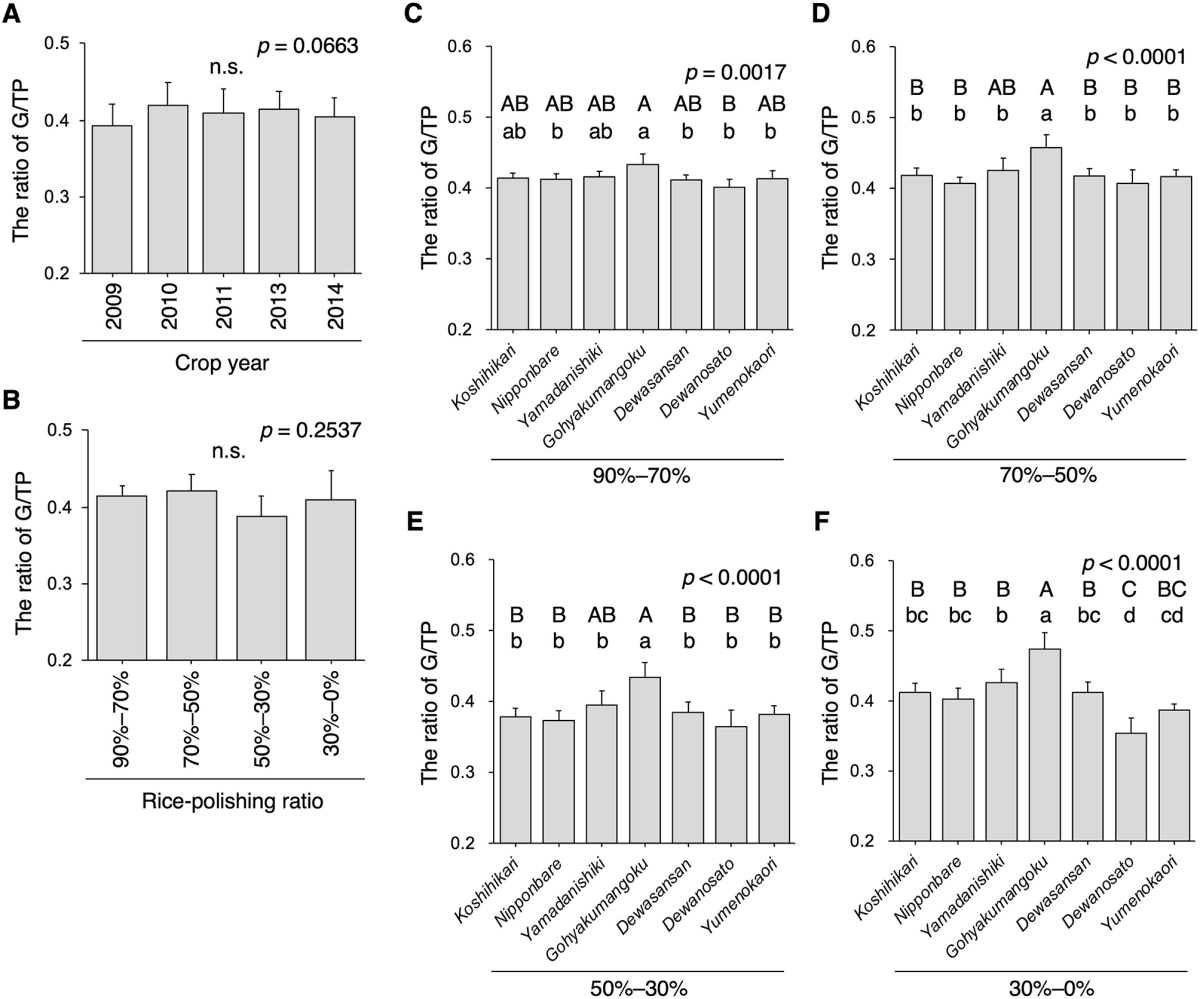
The ratio of glutelin to total protein in rice. (A) The average ratio of glutelin to total protein (G/TP) in white rice grains with a 90% rice-polishing ratio from seven rice varieties is shown (*n* = 28). (B) The average ratio of G/TP in the fractionated rice powder of different rice varieties is shown (*n* = 35). The ratio of G/TP of rice-polishing ratios of 90–70% (C), 70–50% (D), 50–30% (E), and 30–0% (F) (*n* = 5, as crop year). Data containing different capital and small letters indicate significance by Tukey–Kramer HSD test at *p* < 0.01 and *p* < 0.05, respectively. Nonsignificance between groups is indicated as “n.s.” The *p*-value by ANOVA is shown in the inset

The G/TP ratio was the highest in *Gohyakumangoku* among the other cultivars for any rice powder fraction or harvest years (Fig. 4 and Supplementary Fig. 1), strongly suggesting that *Gohyakumangoku* has a higher G/TP ratio in sake rice. Interestingly, the G/TP ratio in *Yamadanishiki* was relatively high among the investigated rice varieties (Fig. 4). but the ratio was lowest in *Dewanosato*. Despite having lower crude protein contents (Fig. 2), *Yamadanishiki* and *Dewanosato* presented markedly varying G/TP ratios. The G/TP ratio differed greatly among rice varieties, particularly in the rice’s central region (Fig. 4C–F). ANOVA indicated no significant differences among the fractionated rice flours but a slightly lower G/TP ratio for the rice-polishing ratio of 50–30% (Fig. 4B). Evaluation of the average G/TP ratio for every rice variety revealed the least G/TP ratio in the 50–30% fraction, with only the *Dewanosato* cultivar showing the least value in the 30–0% fraction. To examine the effect of crop year on the G/TP ratio, we compared the ratios by crop year. No significant differences were observed by ANOVA (Fig. 4A); however, a significant difference was detected between 2009 (lower temperature in summer (Takahashi et al. 2019) and 2010 (hotter temperature in summer) based on paired-*t*-test (*p* < 0.01).

### The Ratio of Prolamin/Total Protein

**Fig. 5:**
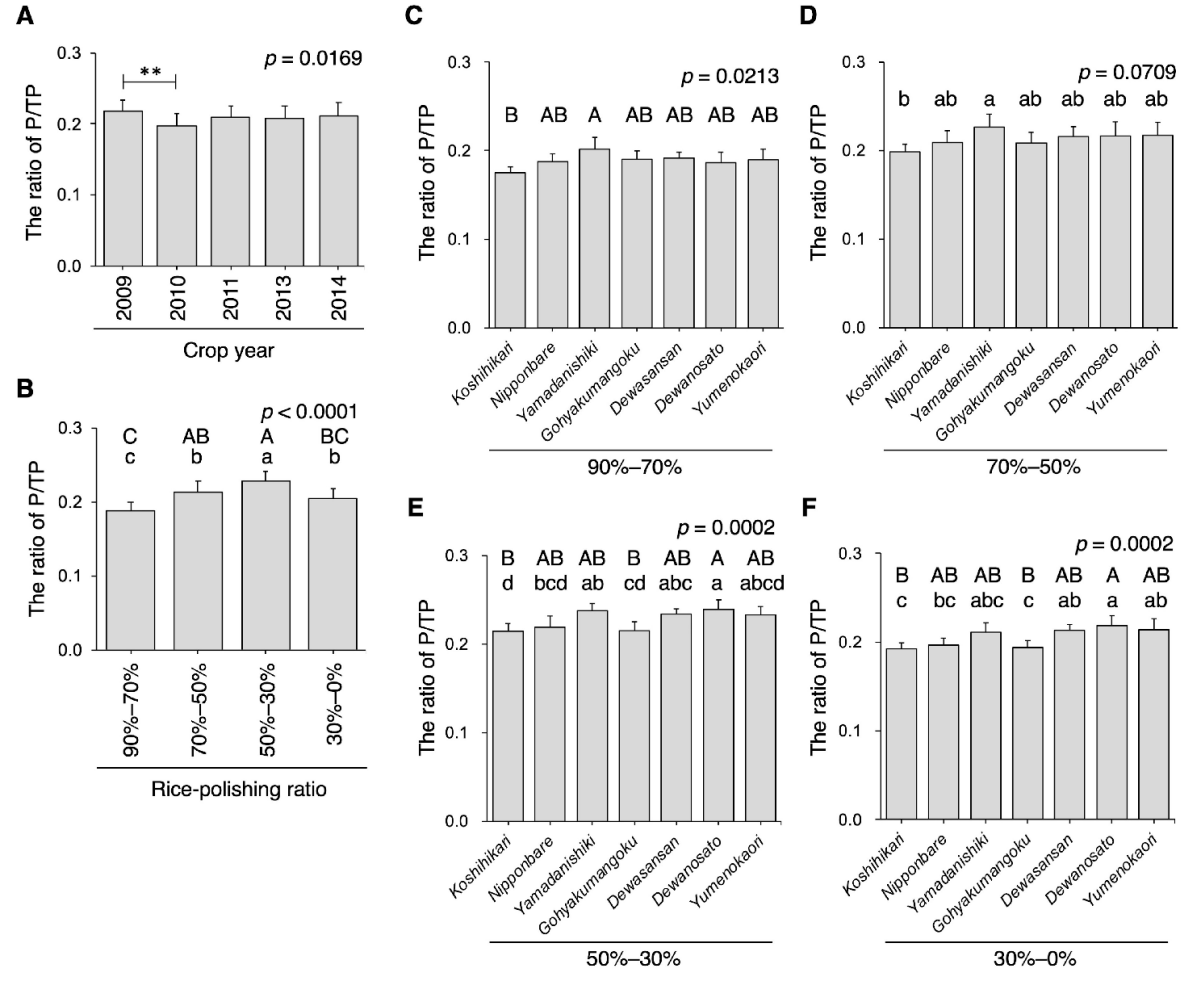
The ratio of prolamin to total protein in rice. (A) The average ratio of prolamin to total protein (P/TP) in white rice grains with a 90% rice-polishing ratio from seven rice varieties is shown (*n* = 28). (B) The average ratio of P/TP in the fractionated rice powders of different rice varieties is shown (*n* = 35). The ratio of P/TP of rice-polishing ratios of 90–70% (C), 70–50% (D), 50–30% (E), and 30–0% (F) (*n* = 5, as crop year). Data containing different capital and small letters indicate significance by Tukey–Kramer HSD test at *p* < 0.01 and *p* < 0.05, respectively. Nonsignificance between groups is indicated as “n.s.” The *p*-value by ANOVA is shown in the inset

ANOVA revealed a significant difference among crop years regarding the P/TP ratio, with a clear difference observed (*p* < 0.01) between 2009 and 2010—higher P/TP ratios in 2010 and lower P/TP ratios in 2009 (Fig. 5A). Prolamin gene expression in rice grains decreases when grains develop at high temperatures (Lin et al. 2010; Yamakawa and Hakata 2010; Yamakawa et al. 2007). Prolamin protein levels were found to decrease in the 90% rice-polishing ratio of *Yamadanishiki* (Ashida et al. 2013) when grains developed at high temperatures—findings that corroborated the present results. The rice flour fractions exhibited markedly varying P/TP ratios (Fig. 5B), with a rice-polishing ratio of 90–70% having the lowest value and a fraction of 50–30% having the highest value. In addition, for the rice flour fractions, the average calculated for each rice variety showed results similar to the whole average (Fig. 5B and Supplementary Fig. 2)—the P/TP ratio was low in the 90–70% rice-polishing ratio fraction and high in the 50–30% rice-polishing ratio fraction, regardless of the rice variety. This finding suggests that the proportion of prolamin to TPs differs according to the spatial distribution of rice grains and that this feature is common to Japonica species. Unlike the G/TP ratio, the P/TP ratio showed no clear difference between rice varieties in the rice grains’ outer region (Fig. 5C–D), although a significant difference was observed in the central region (Fig. 5E–F). In *Koshihikari*, the P/TP ratio tended to be lower than that in the other rice varieties (Fig. 5C–F).

### The Ratio of Glutelin/Prolamin

**Fig. 6:**
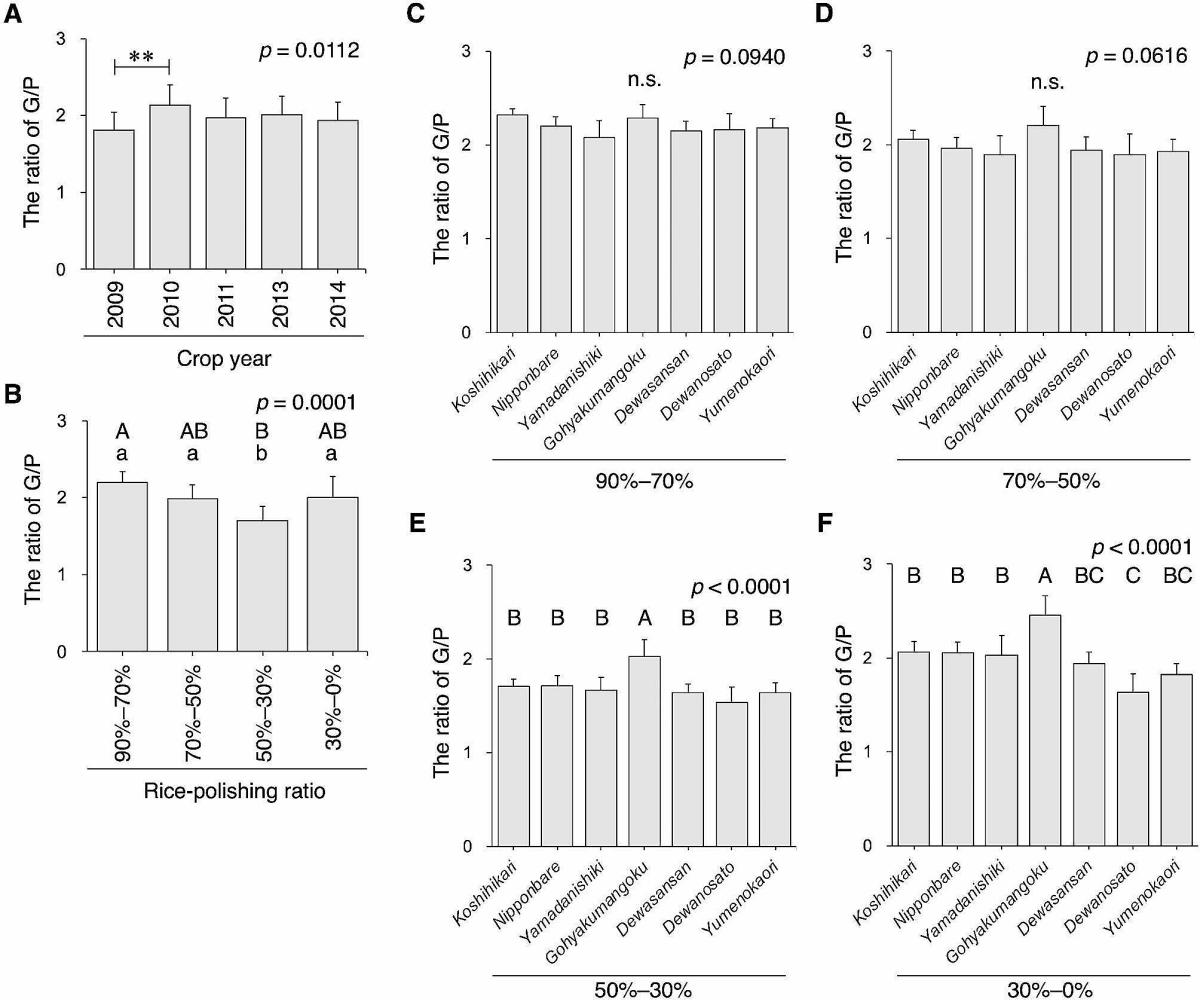
The ratio of glutelin to prolamin in rice. (A) The average ratio of glutelin to prolamin (G/P) in white rice grains with a 90% rice-polishing ratio from seven rice varieties is shown (*n* = 28). (B) The average ratio of G/P in the fractionated rice powders of different rice varieties is shown (*n* = 35). The ratio of G/P of rice-polishing ratios of 90–70% (C), 70–50% (D), 50–30% (E), and 30–0% (F) (*n* = 5, as crop year). Data containing different capital letters indicate significance by Tukey–Kramer HSD test at *p* < 0.01. Nonsignificance between groups is indicated as “n.s.” The *p*-value is shown in the inset. The double asterisk shows statistical significance by Tukey–Kramer HSD test with *p* < 0.01

Based on the P/TP ratio (Fig. 5B), the G/P ratio was high in the rice-polishing ratio fractions of 90–70% and low in the rice-polishing ratio fractions of 50–30%, regardless of the cultivar, except for *Gohyakumangoku*, in which the G/P ratio at the 30–0% fraction was the highest among all fractions (Fig. 6 and Supplementary Fig. 3) due to highly expressed glutelin in the rice grains’ central region (Fig. 4F). *Gohyakumangoku* had a higher G/P ratio than the other varieties, especially in the rice’s central region, in the fractions with a rice-polishing ratio of below 70% (Fig. 6D–F). This finding corroborated a previous result in which the rice-polishing ratio of 70% white rice exhibited a higher PB-II/PB-I ratio in *Gohyakumangoku* owing to lower prolamin content (Kizaki et al. 1993). The 2010-cropped rice had a significantly higher G/P ratio than the 2009-cropped rice (*p* < 0.01). The PB-II/PB-I ratio was found to increase in the 90% rice-polishing ratio of *Yamadanishiki* when grains developed at high temperatures (Ashida et al. 2013). Our findings support those of previous studies. Although the G/P ratio in the rice grain’s outer region varied insignificantly among the rice varieties (Fig. 6C–D), the ratio in the central region varied based on rice variety (Fig. 6E–F), which could be due to the characteristics of the G/TP ratio inherent in rice varieties. Notably, the difference in the G/P ratio among rice varieties was stronger in the rice grains’ central region (Fig. 6E–F).

### Ratio of Non-glutelin and Non-prolamin Proteins

**Fig. 7:**
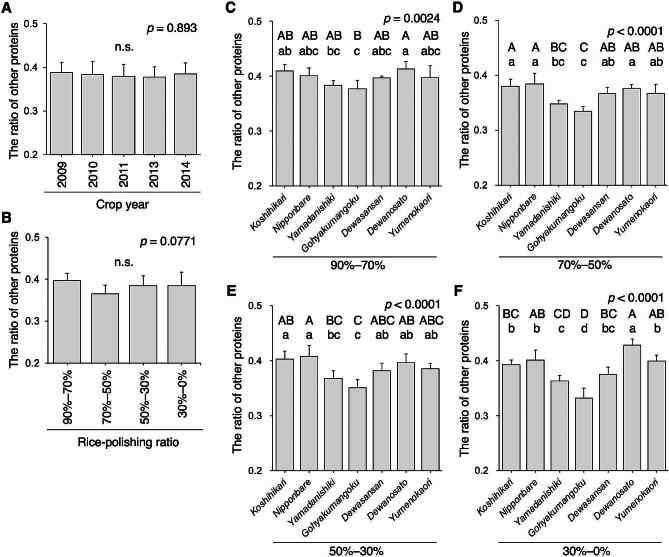
The ratio of non-glutelin and non-prolamin proteins in rice. (A) The average ratio of non-glutelin and non-prolamin proteins (TP–G–P) in white rice grains with a 90% rice-polishing ratio for the seven rice varieties is shown (*n* = 28). (B) The average ratio of TP–G–P in the fractionated rice powder of different rice varieties is shown (*n* = 35). The ratio of TP–G–P of rice-polishing ratios of 90–70% (C), 70–50% (D), 50–30% (E), and 30–0% (F) (*n* = 5, as crop year). Data containing different capital letters indicate significance by Tukey–Kramer HSD test at *p* < 0.01. Nonsignificance between groups is indicated as “n.s.” The *p*-value is shown in the inset. The double asterisk shows statistical significance by Tukey–Kramer HSD test with *p* < 0.01

The proportion of non-glutelin and non-prolamin proteins (TP–G–P) was calculated by subtracting the G/TP and P/TP ratios from 1. This value contains PB-I-constituting proteins such as 10-kDa and 16-kDa prolamins derived from RP10 and RP16 genes (Masumura et al. 1989b; Mitsukawa et al. 1999), respectively, as well as PB-II-constituting proteins, such as 26-kDa globulin (Iida et al. 1998) and pro-glutelin. Non-glutelin and non-prolamin proteins varied insignificantly across crop years (Fig. 7A). Among the rice flour fractions, a higher value in the 90–70% fraction and a lower value in the 70–50% fraction were observed, but this was not statistically significant (Fig. 7B, *p* = 0.0771). The difference in the rice variety was remarkable, with the lowest in *Gohyakumangoku* compared with the other cultivars for any rice powder fraction, followed by *Yamadanishiki* (Fig. 7C–F). The higher G/TP ratio in these cultivars compared to other rice varieties supports this result (Fig. 4C–F). While the difference in the TP–G–P ratio among the rice varieties was pronounced in the rice grains’ central region (Fig. 7C–F), the presence of many clear and rather strong bands located beyond approximately 35-kDa in the rice-polishing ratio fractions of 30–0% suggests that certain specific proteins are abundant in this region (Fig. 3).

## Discussion

Prolamin and glutelin are major protein species in rice grains, as they accumulate in the protein bodies PB-I and PB-II, respectively. However, the spatial patterns in rice grains, especially in the endosperm tissue, and differences in rice varieties remain unclarified. In this study, we addressed the spatial, quantity, and quality differences in rice proteins. Although *Yamadanishiki* and *Dewanosato* presented almost the same crude protein content (Figs. 1 and 2), their G/TP and G/P ratios in the central region of rice grains differed markedly (Figs. 4 and 6), suggesting that the “protein quantity” of both cultivars was comparable, but “protein quality” was differed considerably. Enzymes can readily digest glutelin accumulated in PB-II, indicating that glutelin in steamed rice is vulnerable to proteases and peptidases in rice *koji* (Hashizume et al. 2006, 2007; Iemura et al. 1996; Kizaki et al. 1993; Takahashi et al. 2012). In contrast, prolamin accumulated in PB-I is resistant to digestive enzymes (Tanaka et al. 1975). Glutelin is the product of several glutelin genes (GluA, GluB, GluC, and GluD) (Kawakatsu et al. 2008; Kusaba et al. 2003; Masumura et al. 1989a; Mitsukawa et al. 1998; Qu et al. 2002; Takaiwa et al. 1987, 1991; Takaiwa and Oono 1991) and the amino acid sequence considerably differs among glutelin subfamilies, because glutelin contains the variable region in its amino acid sequence (Kawakatsu et al. 2008; Khan et al. 2008). These facts indicate that protein-degradative nitrogen compounds, such as oligopeptides and amino acids, from these rice varieties may differ in the Japanese sake-making process, that is, mash (*moromi*) and/or sake final products. Differences in nitrogen-containing compounds in sake mash prepared using either *Yamadanishiki* or *Dewanosato* must be explored.

Interestingly, the G/TP and G/P ratios in rice grains dramatically varied based on rice variety, particularly in the rice grains’ central region (Figs. 4 and 6), suggesting that the genetic background of rice strongly reflects the glutelin protein composition. In our previous study, we indicated that the protein expression of GluB-1, a member of the GluB subfamily, was slightly elevated in *Gohyakumangoku*, even in the rice grains’ central region, as assessed using immunodetection assays (Takahashi et al. 2019). GluB-1 protein expression levels among rice varieties may affect the versatility of G/TP and G/P ratios (Figs. 4 and 6). Glutelin molecular species highly expressed in *Gohyakumangoku* require further exploration. Addressing the differences in the expression of each glutelin protein among rice varieties is a crucial area for future research, and it currently constitutes our ongoing focus. Uncovering the differences in the estimated oligopeptides and amino acids when comparing rice varieties is necessary. Although lower levels of prolamin were previously implicated in higher G/P ratios in *Gohyakumangoku* compared to other rice varieties at a 70% rice-polishing ratio (Kizaki et al. 1993), in the present study, the P/TP ratio of *Gohyakumangoku* did not differ significantly from that of other rice varieties, but this variety presented a markedly higher G/TP ratio. Therefore, the higher G/P ratio of *Gohyakumangoku* may have resulted from its higher G/TP ratio.

Unlike glutelin, the difference in prolamin was not pronounced among rice varieties (Fig. 5C–F). *Koshihikari*, a longstanding popular table rice variety in Japan since the 1970s (Kobayashi et al. 2018) due to its sticky texture, had a considerably lower P/TP ratio. This suggests that the P/TP ratio may be involved in the hardness of steamed rice (Anzawa et al. 2006) and palatability of table rice (Takebe et al. 1996), as considered previously. However, it should be noted that this study used a single district for each rice variety, and *Koshihikari* experienced a higher average temperature for one month after rice heading than the other varieties (Takahashi et al. 2019), which may be partly due to earlier rice heading date of the variety (Takahashi et al. 2022) and slightly warm climate of the district chosen. Therefore, prolamin gene expression for *Koshihikari* may have decreased due to high temperatures during the rice developmental stage (Lin et al. 2010; Yamakawa and Hakata 2010; Yamakawa et al. 2007). To define the difference in the P/TP ratio among rice varieties further, experiments will be required to determine the P/TP ratio of rice varieties, including *Koshihikari*, using rice grown under same temperature conditions. Importantly, the P/TP ratio varied significantly among rice flour fractions (Fig. 5B), indicating that the P/TP ratio varied in a spatially dependent manner in rice grains. All rice varieties in this study showed that the P/TP ratio was the lowest at a rice-polishing ratio of 90–70% and the highest at a fraction of 50–30% (Supplementary Fig. 2). The relatively higher proportion of prolamin to TP can confer hardness and prevent stickiness on the surface of the 50% polished steamed rice. We demonstrated that crude protein content in the rice powder fractions differed considerably among the rice varieties (Fig. 2). Herein, we used seven rice cultivars harvested over five cropping years from a single production district. Generally, it is well established that crude protein content in rice can vary depending not only on the rice variety but also on various growth conditions, including nitrogen fertilizer application (Ning et al. 2010; Song et al. 2012). Thus, rigorous comparison of crude protein content among rice cultivars ought to be difficult. We confirmed that the average crude protein content in white rice with a 70% rice-polishing ratio of the seven rice cultivars used in this study was similar to that of *Koshihikari*, *Nipponbare*, *Yamadanishiki*, *Gohyakumangoku*, and *Dewasansan*, which were cropped from 2002 to 2019 (*Koshihikari*, *Nipponbare*, *Yamadanishiki*, and *Gohyakumangoku* were cropped in several different districts), and *Dewanosato* and *Yumenokaori*, which were cropped from 2006 to 2019 [(Takahashi et al. 2021a, b)] data not shown). However, the average crude protein content of *Dewasansan* and *Yumenokaori* used in this study was slightly higher than the previous results (Takahashi et al. 2021a). Therefore, rigorous studies, in which rice plants were grown under the same conditions, should be conducted for precise comparison of the crude protein content among rice varieties.

Finally, the rice-polishing ratio fractions of 30–0% presented many clear and strong bands (Fig. 3). Certain proteins had higher expression levels in the rice grains’ central region than in other spatial regions, such as GluC-1, as reported previously (Takahashi et al. 2019). When brewing *daiginjo*, a premium *ginjo* sake with white rice polished below 50%, the mash should contain peptides and amino acids from proteins that are highly abundant in the rice grains’ central region. Uncovering these proteins will provide valuable insights into the field of brewing science. For effective evaluation of rice grain nitrogen levels and quality, the protein composition ratio should be considered, especially in the rice grains’ central region alongside the crude protein content.

### Electronic Supplementary Material

Below is the link to the electronic supplementary material.

Supplementary Material 1

## Data Availability

All data generated or analyzed during this study are included in this published article

## Abbreviations

ANOVA: analysis of variance
CBB: Coomassie brilliant blue
ER: endoplasmic reticulum
G/P: glutelin/prolamin
G/TP: glutelin/total protein
NRIB: National Research Institute of Brewing
PB: Protein Body
P/TP: prolamin/total protein
SDS-PAGE: sodium dodecyl sulfate-poly acrylamide gel electrophoresis

## References

[CR1] Anzawa Y, Katsumata K, Kataoka A (2006). Hardness and digestibility of steamed rice for sake brewing. J Brew Soc Jpn.

[CR2] Ashida K, Araki E, Maruyama-Funatsuki W, Fujimoto H, Ikegami M (2013). Temperature during grain ripening affects the ratio of type-II/type-I protein body and starch pasting properties of rice (Oryza sativa L). J Cereal Sci.

[CR3] Furukawa S, Mizuma T, Yanagiuchi T, Kiyokawa Y, Wakai Y (2000). Dissolution of protein from rice used for sake brewing in sake mash. J Brew Soc Jpn.

[CR5] Hashizume K, Okuda M, Sakurao S, Numata M, Koseki T, Aramaki I, Kumamaru T, Sato H (2006). Rice protein digestion by sake koji enzymes: comparison between steamed rice grains and isolated protein bodies from rice endosperm. J Biosci Bioeng.

[CR4] Hashizume K, Okuda M, Numata M, Zhou Y, Koseki T (2007). Characterization of peptides generated in proteolytic digest of steamed rice grains by sake koji enzymes. J Biosci Bioeng.

[CR6] Iemura Y, Ohta Y, Hara S (1995). Effects of rice varieties and polishing ratio on the dissolution of protein from steamed rice– studies on nitrogen compounds in sake brewing (part 1). J Brew Soc Jpn.

[CR7] Iemura Y, Kataoka K, Hara S (1996). Effects of the polishing ratio of rice on nitrogen content of sake mash– studies on nitrogen compounds in sake brewing (part 2). J Brew Soc Jpn.

[CR8] Iemura Y, Takahashi T, Yamada T, Furukawa K, Hara S (1999). Properties of TCA-insoluble peptides in kimoto (traditional seed mash for sake brewing) and conditions for liberation of the peptides from rice protein. J Biosci Bioeng.

[CR9] Iemura Y, Yamada T, Takahashi T, Furukawa K, Hara S (1999). Properties of the peptides liberated from rice protein in sokujo-moto. J Biosci Bioeng.

[CR10] Iida S, Miyahara K, Nishio T (1998). Rice mutant lines lacking α-globulin. Breed Sci.

[CR11] Iwano K, Ito T, Nakazawa N (2005). Correlation analysis of a sensory evaluation and the chemical components of ginjyo-shu. J Brew Soc Jpn.

[CR12] Kanauchi M (2013) SAKE alcoholic beverage production in Japanese food industry IntechOpen Limited, London, SW7 2QJ. UNITED KINGDOM

[CR13] Kawakatsu T, Takaiwa F (2010). Cereal seed storage protein synthesis: fundamental processes for recombinant protein production in cereal grains. Plant Biotechnol J.

[CR14] Kawakatsu T, Yamamoto MP, Hirose S, Yano M, Takaiwa F (2008). Characterization of a new rice glutelin gene GluD-1 expressed in the starchy endosperm. J Exp Bot.

[CR15] Khan N, Katsube-Tanaka T, Iida S, Yamaguchi T, Nakano J, Tsujimoto H (2008). Diversity of rice glutelin polypeptides in wild species assessed by the higher-temperature sodium dodecyl sulfate-polyacrylamide gel electrophoresis and subunit-specific antibodies. Electrophoresis.

[CR16] Kizaki Y, Inoue Y, Okazaki N, Kobayashi S (1991). Isolation and determination of protein bodies (PB-I and PB-II) in polished rice endosperm. J Brew Soc Jpn.

[CR17] Kizaki Y, Ohara A, Henmi A, Aramaki I, Kobayashi S, Okazaki N (1993). Difference in protein body components among rice cultivars used for sake brewing. J Brew Soc Jpn.

[CR18] Kobayashi A, Hori K, Yamamoto T, Yano M (2018). Koshihikari: a premium short-grain rice cultivar– its expansion and breeding in Japan. Rice.

[CR19] Kondo M, Nozoe T (1993). Relationship between rice grain composition and eating quality. Tohoku Agric Res.

[CR20] Kumamaru T, Uemura Y, Inoue Y, Takemoto Y, Siddiqui SU, Ogawa M, Hara-Nishimura I, Satoh H (2010). Vacuolar processing enzyme plays an essential role in the crystalline structure of glutelin in rice seed. Plant Cell Physiol.

[CR21] Kusaba M, Miyahara K, Iida S, Fukuoka H, Takano T, Sassa H, Nishimura M, Nishio T (2003). Low glutelin content1: a dominant mutation that suppresses the glutelin multigene family via RNA silencing in rice. Plant Cell.

[CR22] Lin CJ, Li CY, Lin SK, Yang FH, Huang JJ, Liu YH, Lur HS (2010). Influence of high temperature during grain filling on the accumulation of storage proteins and grain quality in rice (Oryza sativa L). J Agric Food Chem.

[CR23] Martin M, Fitzgerald MA (2002). Proteins in rice grains influence cooking properties!. J Cereal Sci.

[CR24] Masumura T, Kidzu K, Sugiyama Y, Mitsukawa N, Hibino T, Tanaka K, Fujii S (1989a) Nucleotide sequence of a cDNA encoding a major rice glutelin. Plant Mol Biol 12(6):723–725. 10.1007/BF00044163 10.1007/BF00044163 24271205

[CR25] Masumura T, Shibata D, Hibino T, Kato T, Kawabe K, Takeba G, Tanaka K, Fujii S (1989). cDNA cloning of an mRNA encoding a sulfur-rich 10 kDa prolamin polypeptide in rice seeds. Plant Mol Biol.

[CR26] Matsue Y, Odahara K, Hiramatsu M (1996). Studies on palatability of rice in Northern Kyushu: VII. Locational differences in palatability of rice and its related factors. Jpn J Crop Sci.

[CR27] Mitsukawa N, Hayashi H, Yamamoto K, Kidzu K, Konishi R, Masumura T, Tanaka K (1998). Molecular cloning of a novel glutelin cDNA from rice seeds. Plant Biotechnol.

[CR28] Mitsukawa N, Konishi R, Kidzu K, Ohtsuki K, Masumura T, Tanaka K (1999). Amino acid sequencing and cDNA cloning of rice seed storage proteins, the 13 kDa prolamins, extracted from type I protein bodies. Plant Biotechnol.

[CR29] Mizuma T, Furukawa S (2004). Characteristics of low-glutelin rice for sake brewing. J Brew Soc Jpn.

[CR30] Mizuma T, Furukawa S, Kiyokawa Y (2002). Characteristics of low-glutelin rice for sake brewing: studies on rice for sake brewing. Seibutsu-Kogaku Kaishi.

[CR31] Nagamine A, Matsusaka H, Ushijima T, Kawagoe Y, Ogawa M, Okita TW, Kumamaru T (2011). A role for the cysteine-rich 10 kDa prolamin in protein body I formation in rice. Plant Cell Physiol.

[CR32] Ning H, Qiao J, Liu Z, Lin Z, Li G, Wang Q, Wang S, Ding Y (2010). Distribution of proteins and amino acids in milled and brown rice as affected by nitrogen fertilization and genotype. J Cereal Sci.

[CR33] Qu Q, Wei L, Satoh H, Kumamaru T, Ogawa M, Takaiwa F (2002). Inheritance of alleles for glutelin alpha-2 subunit genes in rice and identification of their corresponding cDNA clone. Theor Appl Genet.

[CR34] Saito Y, Shigemitsu T, Yamasaki R, Sasou A, Goto F, Kishida K, Kuroda M, Tanaka K, Morita S, Satoh S, Masumura T (2012). Formation mechanism of the internal structure of type I protein bodies in rice endosperm: relationship between the localization of prolamin species and the expression of individual genes. Plant J.

[CR35] Song Y-J, Choi I-Y, Sharma PK, Kang C-H (2012). Effect of different nitrogen doses on the storage proteins and palatability of rice grains of primary and secondary rachis branches. Plant Prod Sci.

[CR36] Takahashi K, Kohno H (2016). Different polar metabolites and protein profiles between high- and low-quality Japanese *ginjo* sake. PLoS ONE.

[CR41] Takahashi K, Tokuoka M, Kohno H, Sawamura N, Myoken Y, Mizuno A (2012). Comprehensive analysis of dipeptides in alcoholic beverages by tag-based separation and determination using liquid chromatography/electrospray ionization tandem mass spectrometry and quadrupole-time-of-flight mass spectrometry. J Chromatogr A.

[CR37] Takahashi K, Kohno H, Kanabayashi T, Okuda M (2019). Glutelin subtype-dependent protein localization in rice grain evidenced by immunodetection analyses. Plant Mol Biol.

[CR39] Takahashi K, Okuda M, Joyo M, Numata M, Bao H, Kohno H (2021). Comparative analysis of sake rice varieties utilizing earlier-period-rice analysis data collected from 2002–2019 crop years. J Brew Soc Jpn.

[CR40] Takahashi K, Okuda M, Joyo M, Numata M, Bao H, Kohno H (2021). Time course study for elucidating annual variation of the brewing properties of sake rice utilizing earlier-period-rice analysis data collected from 2002–2019 crop years. J Brew Soc Jpn.

[CR38] Takahashi K, Okuda M, Joyo M, Numata M (2022). Effects of temperature conditions after rice heading on enzyme digestibility (Brix) of steamed rice for the 2002–2019 crop years. J Brew Soc Jpn.

[CR43] Takaiwa F, Oono K (1991). Genomic DNA sequences of two new genes for new storage protein glutelin in rice. Jpn J Genet.

[CR42] Takaiwa F, Kikuchi S, Oono K (1987). A rice glutelin gene family — a major type of glutelin mRNAs can be divided into two classes. Mol Gen Genet.

[CR44] Takaiwa F, Oono K, Wing D, Kato A (1991). Sequence of three members and expression of a new major subfamily of glutelin genes from rice. Plant Mol Biol.

[CR45] Takebe M, Oikawa T, Matsuno K, Shimizu E, Yoneyama T (1996). Influence of nitrogen application on the contents of glutelin and prolamin of polished rice grains (Oryza sativa L). Jpn J Soil Sci Plant Nutr.

[CR47] Tanaka Y, Hayashida S, Hongo M (1975). The relationship of the feces protein particles to rice protein bodies. Agric Biol Chem.

[CR46] Tanaka K, Sugimoto T, Ogawa M, Kasai Z (1980). Isolation and characterization of two types of protein bodies in the rice endosperm. Agric Biol Chem.

[CR48] Wakai Y, Mizuma T, Miyazaki N, Nagano T, Yanagiuchi T (1997). Effect of properties of rice on suitability for sake brewing. Seibutsu-Kogaku Kaishi.

[CR49] Wang Y, Zhu S, Liu S, Jiang L, Chen L, Ren Y, Han X, Liu F, Ji S, Liu X, Wan J (2009). The vacuolar processing enzyme OsVPE1 is required for efficient glutelin processing in rice. Plant J.

[CR50] Xu JH, Messing J (2009). Amplification of prolamin storage protein genes in different subfamilies of the Poaceae. Theor Appl Genet.

[CR51] Yamagata H, Sugimoto T, Tanaka K, Kasai Z (1982). Biosynthesis of storage proteins in developing rice seeds. Plant Physiol.

[CR52] Yamakawa H, Hakata M (2010). Atlas of rice grain filling-related metabolism under high temperature: joint analysis of metabolome and transcriptome demonstrated inhibition of starch accumulation and induction of amino acid accumulation. Plant Cell Physiol.

[CR53] Yamakawa H, Hirose T, Kuroda M, Yamaguchi T (2007). Comprehensive expression profiling of rice grain filling-related genes under high temperature using DNA microarray. Plant Physiol.

