# Supplementary material for: Spatial Distribution and Characteristics of Protein Content and Composition in Japonica Rice Grains: Implications for Sake Quality
Source: Rice (N Y). 2024 Apr 12;17:26. doi: 10.1186/s12284-024-00708-w (PMC11014839; doi:10.1186/s12284-024-00708-w)

Supplementary Materials

Journal: Rice

Title: Spatial Distribution and Characteristics of Protein Content and Composition in Japonica Rice Grains: Implications for Sake Quality

Authors: Kei Takahashi^1*^, Hiromi Kohno^1^, and Masaki Okuda^1^

Author affiliations:

1, National Research Institute of Brewing, 3-7-1 Kagamiyama, Higashi-hiroshima, Hiroshima, 739-0046, Japan

*Corresponding author. Tel.: +81-82-420-8227; Fax: +81-82-420-8228. E-mail address: [k.takahashi@nrib.go.jp](mailto:k.takahashi@nrib.go.jp)

Supplementary Fig. 1. The ratio of glutelin to total protein in rice grains

The G/TP ratio of the rice-polishing ratios of 90%–70% (closed bars), 70%–50% (dark gray bars), 50%–30% (light gray bars), and 30%–0% (opened bars): n = 5 (2009, 2010, 2011, 2013, 2014)

Supplementary Fig. 2. The ratio of prolamin to total protein in rice grains

The P/TP ratio of the rice-polishing ratios of 90%–70% (closed bars), 70%–50% (dark gray bars), 50%–30% (light gray bars), and 30%–0% (opened bars): n = 5 (2009, 2010, 2011, 2013, 2014)

Supplementary Fig. 3. The ratio of glutelin to prolamin in rice grains

The G/P ratio of the rice-polishing ratios of 90%–70% (closed bars), 70%–50% (dark gray bars), 50%–30% (light gray bars), and 30%–0% (opened bars): n = 5 (2009, 2010, 2011, 2013, 2014)


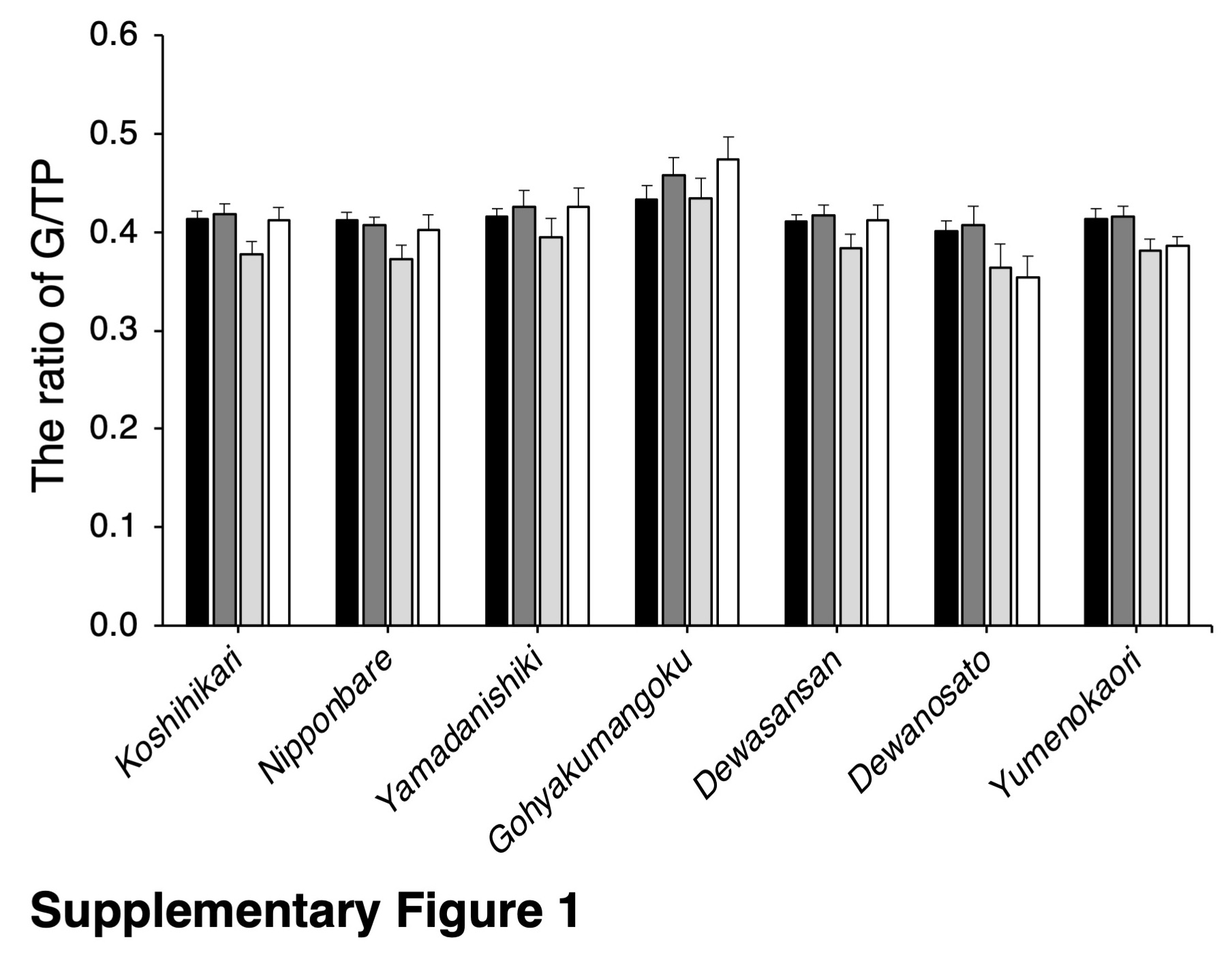


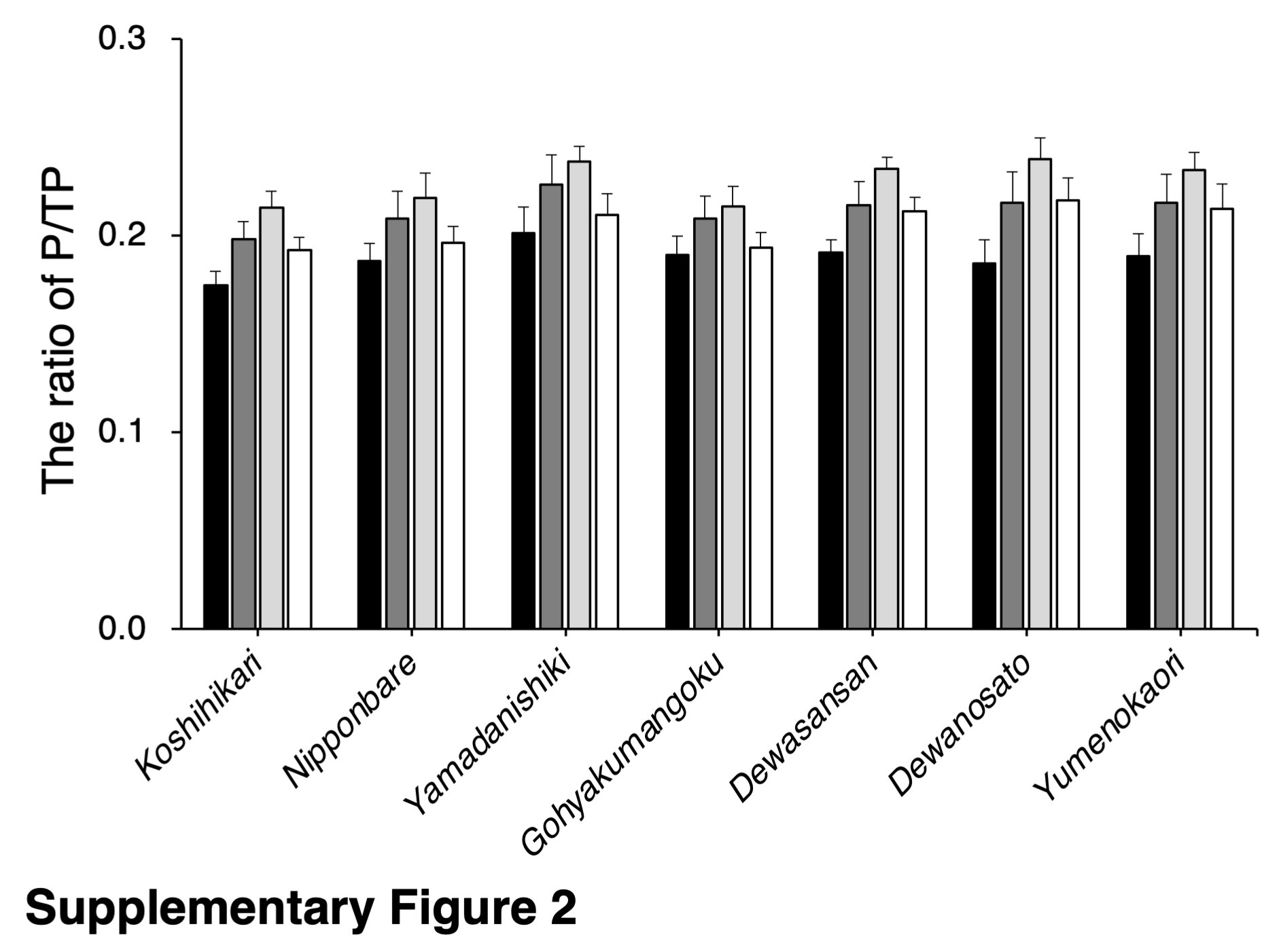


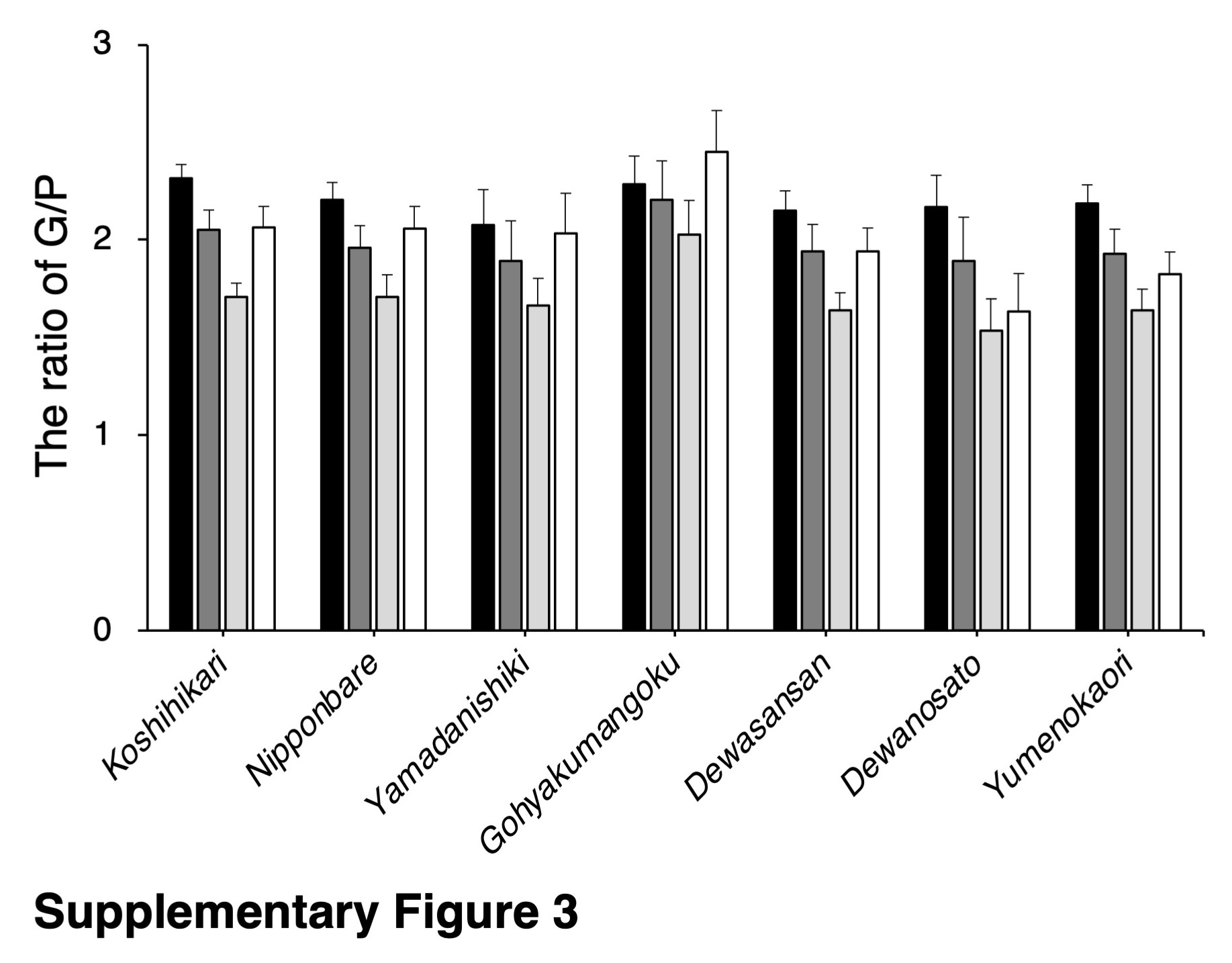

Supplement: Supplementary file 1 — Supplementary Material 1 [file 12284_2024_708_MOESM1_ESM.docx]
